# Aptazyme-embedded guide RNAs enable ligand-responsive genome editing and transcriptional activation

**DOI:** 10.1038/ncomms15939

**Published:** 2017-06-28

**Authors:** Weixin Tang, Johnny H. Hu, David R. Liu

**Affiliations:** 1 Department of Chemistry and Chemical Biology, Harvard University, 12 Oxford Street, Cambridge, Massachusetts 02138, USA; 2 Howard Hughes Medical Institute, Harvard University, 12 Oxford Street, Cambridge, Massachusetts 02138, USA; 3 Broad Institute of MIT and Harvard, Cambridge, Massachusetts 02141, USA

## Abstract

Programmable sequence-specific genome editing agents such as CRISPR-Cas9 have greatly advanced our ability to manipulate the human genome. Although canonical forms of genome-editing agents and programmable transcriptional regulators are constitutively active, precise temporal and spatial control over genome editing and transcriptional regulation activities would enable the more selective and potentially safer use of these powerful technologies. Here, by incorporating ligand-responsive self-cleaving catalytic RNAs (aptazymes) into guide RNAs, we developed a set of aptazyme-embedded guide RNAs that enable small molecule-controlled nuclease-mediated genome editing and small molecule-controlled base editing, as well as small molecule-dependent transcriptional activation in mammalian cells.

## Abstract (web summary)

CRISPR-Cas9 is a powerful technique for manipulating the human genome, however temporal and spatial control of activity would facilitate safer, more selective use. Here the authors incorporate aptazymes into guide RNAs to allow small molecule activation of CRISPR-Cas9.

Clustered regularly interspaced short palindromic repeat (CRISPR)-associated protein 9 (Cas9) is an RNA-programmed endonuclease that enables the efficient, sequence-specific modification of target loci in the human genome^1^,^2^. Catalytically inactive forms of Cas9 fused to transcriptional activator and repressor domains can serve as programmable transcriptional regulators^3^,^4^,^5^,^6^. More recently, we fused a cytidine deaminase, a Cas9 nickase, and a base excision repair inhibitor to engineer ‘base editors’ that introduce C:G to T:A mutations in the human genome without inducing double-stranded DNA breaks^7^. Canonical forms of Cas9 are constitutively active and are not dependent on any metabolite, exogenous ligand, or cell state. The development of Cas9 systems that can be precisely controlled in living cells by exogenous, cell-permeable small molecules would enhance their ability to serve as research tools and as potential therapeutics to treat diseases with a genetic component by increasing their specificity and reducing the likelihood of editing at off-target loci or in non-target tissues^8^,^9^.

Substantial effort has been devoted to the development of controllable genome-editing agents in mammalian cells^10^,^11^. Placing the expression of Cas9 under the control of inducible or tissue-specific promoters provides limited control over genome editing. Manipulation at the transcriptional level, however, offers poor temporal control compared with post-transcriptional and post-translational control, and is difficult to achieve in many cell types due to a lack of tissue-specific promoters. Post-translationally regulated Cas9 variants have been developed in which conditionally inactive Cas9 proteins are expressed and genome editing activity is restored only in the presence of specific stimuli^12^,^13^,^14^,^15^. Controlling the activity of Cas9 to limit the temporal exposure of cells to genome editing activity has resulted in important benefits such as reduced modification of off-target loci^13^.

A complementary and much less-explored strategy to controlling Cas9-mediated genome editing or transcriptional regulation is to develop ligand-responsive guide RNAs. Both nature and researchers have evolved many RNAs that undergo secondary structure changes in the presence of small molecules^16^,^17^, and these principles in theory could be used to engineer ligand-responsive guide RNAs. Aptazymes are ligand-activate self-cleaving ribozymes that contain integrated aptamer domains^18^. Upon binding ligands of interest, aptazymes undergo structural changes that activate an associated ribozyme domain, triggering RNA cleavage.

In this study we developed aptazyme-embedded guide RNAs (agRNAs) that enable small-molecule control of guide RNA structure and genome engineering activity. Different guide RNA architectures were tested and optimized both *in vitro* and in mammalian cells, resulting in the efficient liberation of active guide RNAs in the presence of the triggering molecules. We used agRNAs to achieve theophylline-controlled nuclease-mediated genome editing and base editing, as well as guanine-controlled transcriptional activation in mammalian cells.

## Results

### Blocking guide RNA activity

**Figure 1:**
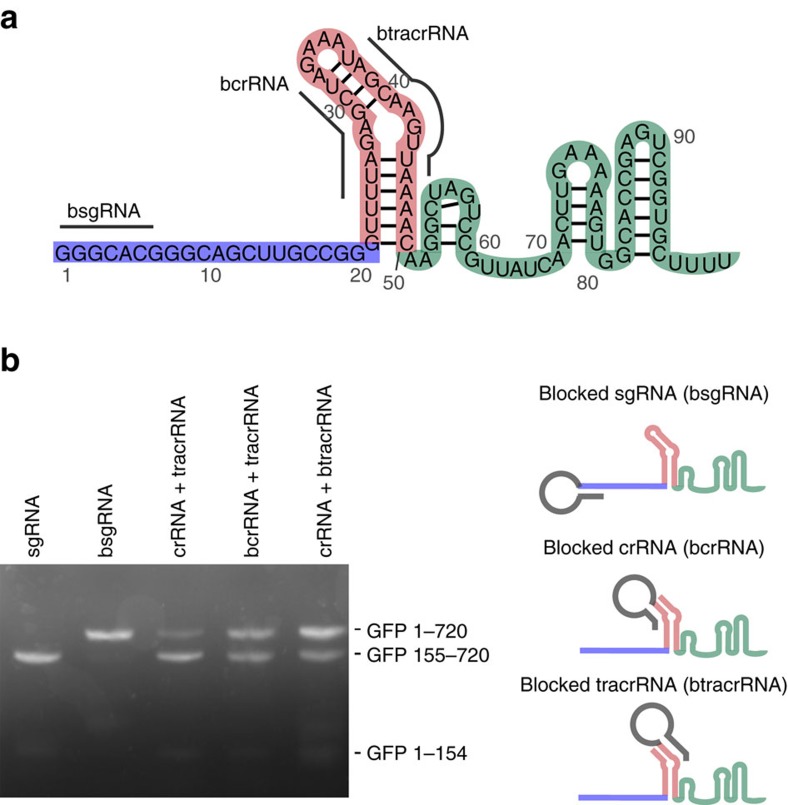
Strategies to abrogate the activity of guide RNAs by appending blocking sequences. (**a**) Schematic representation of the essential regions of the guide RNA that were targeted by candidate blocking sequences. (**b**) *In vitro* activity of unmodified guide RNAs and three blocked guide RNA variants depicted in the lower right. The canonical and blocked sgRNAs were each incubated with the target dsDNA (the full-length GFP gene) in the presence of Cas9 for 1 h at 37 °C, while the crRNA and tracrRNA variants were incubated for an additional 4 h due to the lower overall activities of the corresponding Cas9:guide RNA complexes.

To inactivate a guide RNA in the absence of ligand, we extended the length of the guide RNA to install a ‘blocking sequence’ complementary to an essential region of the guide RNA. We tested two key regions of the *Streptococcus pyogenes* single guide RNA (sgRNA) as the targets of the blocking sequences: (1) the spacer and (2) the annealing region between the crRNA and tracrRNA (Fig. 1a)^1^. The crystal structure of the guide RNA bound to Cas9 suggests that an elongated 5′ end and insertions in the tetraloop should not significantly interfere with guide RNA function^19^,^20^. Therefore, we appended a 42-nucleotide linker followed by a 6-nucleotide (nt) blocking sequence that complements nucleotides 1–6 in the spacer to the 5′ end of the sgRNA (Fig. 1a). We envisioned eventually replacing the linker with a ligand-responsive RNA and chose an elongated linker to test if the size and flexibility of the RNA would disturb intramolecular hybridization of the blocking sequence to the spacer. To block the interaction between the crRNA and tracrRNA, we appended to the 3′ end of the crRNA or to the 5′ end of the tracrRNA the 42-nucleotide linker and a 10-nucleotide blocking sequence that is complementary to the annealing region in the crRNA or tracrRNA, respectively (Fig. 1a).

The blocker-linked guide RNAs were prepared by *in vitro* transcription and incubated *in vitro* with Cas9 protein and a target dsDNA. Target DNA cleavage was followed by gel electrophoresis (Fig. 1b; Supplementary Fig. 1). The activity of the 5′ spacer-blocked sgRNA was very low and no cleavage product could be detected by gel electrophoresis, while cleavage catalysed by the unmodified sgRNA approached 100% efficiency under the same conditions (Fig. 1b; Supplementary Fig. 1). Although the blocked crRNA and tracrRNA were less active than the unmodified guide RNAs, they both resulted in partial cleavage of the target DNA, indicating that the strategy for blocking the crRNA and tracrRNA was not as effective as the use of a spacer-blocked sgRNA (bsgRNA).

Together, these findings demonstrate an effective strategy for using RNA to occlude an essential region of a sgRNA, thereby abrogating Cas9 activity. On the basis of these results, we chose the bsgRNA architecture as the starting point for our efforts to engineer ligand-induced restoration of guide RNA function.

### Guide RNA activation by removal of the blocking sequence

**Figure 2:**
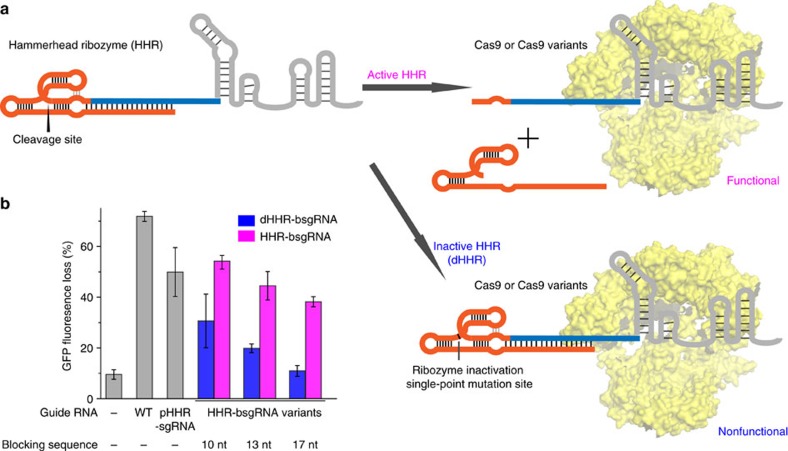
Restoring blocked guide RNA activity with an embedded hammerhead ribozyme in human cells. (**a**) Schematic representation of the hammerhead ribozyme-embedded guide RNA and the expected activities in the presence of Cas9; (**b**) Genome-editing activities of hammerhead ribozyme-embedded guide RNAs in HEK293-GFP cells. The blocking sequence that is complementary to the spacer was fused to the guide RNA through a hammerhead ribozyme (HHR-bsgRNA). The length of the blocking sequence was varied from 10 to 17 nt. In HHR-bsgRNA variants, the hammerhead ribozyme is active and the blocking sequence is removed *in situ* once the guide RNA is transcribed, whereas in dHHR-bsgRNA variants, the hammerhead ribozyme is inactive and the blocking sequence remains appended to the guide RNA. The full activity of HHR-bsgRNA is shown by directly expressing the processed HHR-bsgRNA without the blocking sequence (pHHR-sgRNA). Canonical sgRNA (WT) served as the positive control and cells that were transfected with the Cas9 expression plasmid but no guide RNA plasmid were used as a negative control. The GFP fluorescence loss was quantified by comparing the mean cell fluorescence in transfected cells to that in cells treated with lipids only. Values and error bars reflect mean GFP fluorescence loss and the s.d. of three biological replicates.

Next, we developed molecular strategies to restore guide RNA activity by removing the blocking sequence *in situ*. We integrated a fast-cleaving hammerhead ribozyme^21^ with low magnesium dependence between the blocking sequence and the guide RNA (HHR-bsgRNA, Fig. 2a). Once transcribed, the hammerhead ribozyme should spontaneously fold and undergo self-cleavage to remove most of the ribozyme as well as the blocking sequence. Self-cleavage transforms the intramolecular base pairs between the spacer and the blocking sequence in the HHR-bsgRNA into less favorable intermolecular interactions, promoting the release of the blocking sequence from the guide RNA. The post-cleavage sgRNA contained 11 extra nucleotides at the 5′ end that are remnants from the hammerhead ribozyme and maintained most of its activity compared to the unmodified guide RNA (Fig. 2b). This result establishes that the ribozyme self-cleavage of the blocking region restores sgRNA activity.

To compare the activities of the guide RNAs with the blocking region either present or removed, we constructed a non-cleaving ‘dead’ variant of HHR-bsgRNA (dHHR-bsgRNA) containing a single A to G point mutation in the catalytic loop of the hammerhead ribozyme. To optimize the restoration of guide RNA activity by self-cleavage of the hammerhead ribozyme, we varied the length of the blocking sequence. We constructed three sets of HHR-bsgRNAs and dHHR-bsgRNAs with blocking sequences of 10 to 17 nucleotides. The genome editing activities of these sgRNA variants in the presence of Cas9 in human embryonic kidney cells harboring genomic GFP genes (HEK293-GFP) were assayed by targeting the endogenous GFP locus (Fig. 2a).

As expected, the activities of both non-cleaving blocked guide RNAs (dHHR-bsgRNAs) and self-cleaving blocked guide RNAs (HHR-bsgRNAs) decreased when the length of the blocking sequence increased (Fig. 2b). We chose a 17-nucleotide blocking sequence for further engineering because it resulted in minimal background activity in the off state that was comparable to a negative control lacking any guide RNA, but showed substantial restoration of activity once the blocking sequence was removed by ribozyme self-cleavage (Fig. 2b).

### Theophylline-dependent genome editing

**Figure 3:**
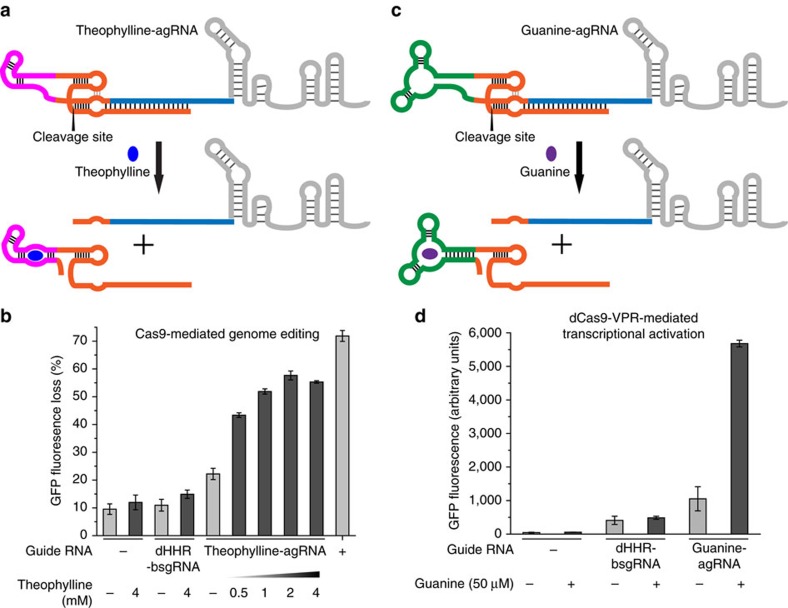
Small molecule-controlled genome editing and transcriptional activation enabled by agRNAs in human cells. (**a**) Schematic representation of the activation of theophylline-agRNA in the presence of theophylline. (**b**) Genome-editing activity of theophylline-agRNA in HEK293-GFP cells. Endogenous GFP sites were edited by the Cas9:guide RNA complex upon agRNA activation. Cells that were not transfected with any guide RNA plasmid served as the negative control, while cells transfected with Cas9 and a canonical sgRNA (+) served as a positive control. The GFP fluorescence loss was quantified by comparing the mean cell fluorescence in transfected cells to that in cells treated with lipids only. (**c**) Schematic representation of the activation of guanine-agRNA in the presence of guanine. (**d**) Transcriptional activation activity of guanine-agRNA in the presence and absence of the ligand in HEK293T cells. GFP activation was achieved using dCas9-VPR:guide RNA complex upon agRNA activation. Values and error bars reflect mean GFP fluorescence and the s.d. of three biological replicates.

Next we sought to impart ligand dependence in an HHR-bsgRNA by replacing the hammerhead ribozyme with an aptazyme that contains a ligand-binding aptamer that transduces the presence of a small molecule into ribozyme self-cleavage. We replaced the hammerhead ribozyme with the theophylline-dependent aptazyme to generate theophylline-agRNA^22^,^23^. To ensure that the theophylline-dependent aptazyme uses the same cleavage site as the unmodified ribozyme when fused to the guide RNA, the theophylline-agRNA was transcribed *in vitro* and the cleaved products were analyzed by mass spectrometry. The observed masses were in good agreement with the expected masses for the 5′ fragment and the 3′ fragment (Supplementary Fig. 2), confirming that appending the guide RNA did not alter the cleavage site preference of the hammerhead ribozyme. The genome-editing activity of theophylline-agRNA was further assayed in HEK293-GFP cells (Fig. 3a). While control cells expressing Cas9 but lacking guide RNAs resulted in 9.6% loss of GFP fluorescence, cells that were co-transfected with both Cas9 and theophylline-agRNA exhibited 22% GFP loss in the absence of theophylline (Fig. 3b). This off-state background activity of 12% additional GFP loss is consistent with reports that the activity of the hammerhead ribozyme in the theophylline aptazyme, while substantially decreased, is detectable even in the absence of theophylline^23^. In addition, incorporation of the theophylline aptamer increases the overall bulkiness of the ribozyme domain and the additional steric hindrance may affect the interaction between the blocking sequence and the spacer, resulting in less efficient inhibition.

Importantly, in the presence of theophylline, the activity of the agRNA increased fourfold, resulting in 58% GFP fluorescence loss (48% additional loss beyond the control) (Fig. 3b). By comparison, wild-type Cas9 and a canonical sgRNA resulted in 72% GFP loss (62% loss beyond the control), only 1.3-fold that of the activated agRNA. The activity of the theophylline-agRNA was dependent on the concentration of theophylline, reaching a maximum at 2 mM (Fig. 3b). These results show that ligand-induced agRNA activation can result in genome modification levels that approach those of canonical Cas9:sgRNA complexes.

To rule out the possibility that the observed loss of GFP fluorescence was a consequence of theophylline toxicity rather than genome editing at endogenous GFP sites, cells transfected without any guide RNA plasmid, or with a control plasmid producing the dHHR-bsgRNA were treated with the highest concentration of theophylline used in the above experiments (4 mM). Neither control sample exhibited significant GFP loss in the presence of theophylline compared to the same cells that were not treated with theophylline (Fig. 3b), suggesting that theophylline did not negatively impact cell fluorescence and the observed GFP loss resulted from disruption of the endogenous GFP gene. To confirm that activation of the theophylline-agRNA was due to the ligand-responsive self-cleavage of the aptazyme, rather than to binding of theophylline to the aptamer domain without cleavage, we installed the A to G mutation that inactivates the hammerhead ribozyme into the theophylline-agRNA while maintaining theophylline binding activity^23^ and assayed the genome-editing activity of the resulting ‘dead’ theophylline-agRNA ((d)theophylline-agRNA). This point mutation abolished the ligand response of theophylline-agRNA and resulted in only basal levels of GFP loss in the presence or absence of theophylline, indicating that ribozyme self-cleavage, and not merely ligand binding, is required for agRNA activation (Supplementary Fig. 3). Together, these findings establish that small molecule-induced aptazyme self-cleavage can activate guide RNAs and enable ligand-dependent genome editing.

### Guanine-dependent transcriptional activation

In addition to genome editing, transcriptional regulation is another widely used application of CRISPR technology^4^. To further explore the utility of agRNAs, we sought to develop an agRNA that responds to a different small-molecule ligand and activates the expression of a guide RNA-specified target gene, rather than editing a target gene through nuclease-mediated double-strand break. A previously reported guanine aptazyme^24^ that contains a naturally occurring guanine aptamer^25^ was incorporated into the guide RNA and the resulting guanine-agRNA was tested for its ability to activate transcription of a GFP gene in HEK293T cells (Fig. 3c). The guanine-agRNA targeted the sequence upstream of a modified promoter for the GFP gene and recruited the fusion of catalytically inactive dCas9 with tandem mammalian transcriptional activators VP64-p65-Rta (dCas9-VPR)^4^. In the presence of an active guide RNA, dCas9-VPR initiates transcription of GFP, ultimately resulting in cell fluorescence.

Similar to the theophylline-agRNA, the guanine-agRNA also exhibited detectable off-state activity, resulting in elevated GFP fluorescence in the absence of guanine compared to control cells lacking any guide RNA (Fig. 3d; Supplementary Fig. 4). We attribute the elevated off-state activity to the presence of endogenous guanine, as well as basal self-cleaving activity of the guanine aptazyme in the absence of the small molecule^24^. When guanine was added to media at a concentration of 50 μM, however, a fivefold activation of GFP fluorescence was observed in cells that were transfected with guanine-agRNA expression plasmid. In contrast, no GFP activation was detected in control cells lacking guide RNA or in control cells expressing non-cleaving dHHR-bsgRNA (Fig. 3d; Supplementary Fig. 4). In addition, the non-splicing mutant (d)guanine-agRNA resulted in only a low level of GFP expression that could no longer be activated by guanine (Supplementary Fig. 5). Indeed, increasing the concentration of guanine in the medium from 0 to 200 μM slightly reduced GFP fluorescence (Supplementary Fig. 5), suggesting that guanine might have a modest negative effect on cell fluorescence and the actual activation ratio of the guanine-agRNA could be higher than was calculated from GFP fluorescence.

### Additional optimization of the blocking sequence

Controlling RNA activity by intramolecular blocking sequences has been achieved in other functional RNAs and different architectures of blocking sequences have been explored^23^,^26^. In the ‘riboregulators’ that enable post-transcriptional control of gene expression in *E. coli* by regulating the accessibility of the ribosomal binding site (RBS), blockers containing bulges exhibit stronger activation than blockers that are fully complementary to the RBS, since the bulges destabilize the RNA duplex and facilitate the activation process^26^. Inspired by this observation, we further optimized the blocking sequence in the guanine-agRNA by increasing the blocking sequence to 18 nucleotides and incorporating three bulges.

The newly constructed guanine-agRNA with the optimized blocking sequence exhibited a similar off-state activity to our original guanine-agRNA design but resulted in a slightly higher GFP fluorescence when activated by the presence of guanine (Supplementary Fig. 6). To further generalize this strategy, we tested the guanine-agRNA with three bulges for RFP activation in HEK293T cells with a different spacer sequence that is unrelated to the spacer used to target GFP. Consistent with above results, guanine-dependent RFP activation was observed in the presence of dCas9-VPR with an activation ratio of approximately threefold (Supplementary Fig. 7), suggesting that the agRNA architecture is generally applicable on different spacer sequences as well as different target genes.

### Application of agRNAs to endogenous sites for genome editing

**Figure 4:**
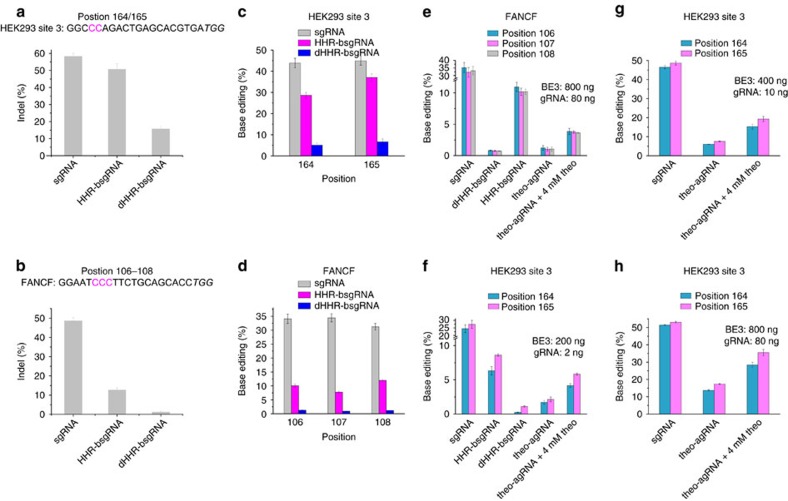
Theophylline-responsive base editing on the HEK-3 and FANCF loci in human cells. (**a**,**b**) Nuclease-mediated indel formation by HHR-bsgRNA and dHHR-bsgRNA on the HEK-3 (**a**) and FANCF (**b**) sites in the presence of Cas9. The protospacer sequences of both sites are shown with the protospacer adjacent motif (PAM) in italics. The base editing target bases are shown in magenta. (**c**,**d**) Base editing by HHR-bsgRNA and dHHR-bsgRNA on the HEK-3 (**c**) and FANCF (**d**) sites in the presence of BE3. (**e**) Theophylline-dependent base editing on the FANCF site. (**f**–**h**) Theophylline-dependent base editing on the HEK-3 site with different doses of agRNA and BE3 plasmids. Values and error bars reflect mean editing percentage and the s.d. of three biological replicates except the dHHR-bsgRNA and HHR-bsgRNA columns in panel e and the theophylline treated samples in **e**–**h**, for which two biological replicates were performed.

To further define the application scope of agRNAs, we tested them on four additional guide RNAs that target endogenous HEK-3, FANCF, EMX-1 and HEK-4 loci in the human genome. To establish the dynamic range of the responsive guide RNAs, we first measured the activities of HHR-bsgRNA and dHHR-bsgRNA with a 17-nucleotide blocking sequence for gene disruption on these four loci. In the presence of Cas9, 1.2 to 16% insertion or deletion (indel) formation was observed with dHHR-bsgRNA, whereas a 3- to 13-fold higher activity could be achieved when the HHR-bsgRNA was introduced (Fig. 4a,b; Supplementary Fig. 8), defining a potential dynamic range for ligand-dependent genome editing at these four endogenous sites.

Base editing is a novel technology that enables programmable single-base pair modification in the human genome without inducing double-stranded DNA breaks^7^,^27^,^28^,^29^. We tested the activities of HHR-bsgRNAs and dHHR-bsgRNA for base editing of endogenous HEK-3, FANCF, EMX-1 and HEK-4 loci in HEK293 cells in the presence of the BE3 base editor^7^. Similar to the case with nuclease-mediated indel formation, base editing occurred with different efficiencies at the four loci (Fig. 4c,d; Supplementary Fig. 8). The active HHR-bsgRNAs resulted in five- to eightfold higher levels of base editing than the inactive dHHR-bsgRNAs on all four sites (Fig. 4c,d; Supplementary Fig. 8), defining the potential dynamic range for ligand-activated base editing. Encouraged by these observations, we engineered the theophylline-responsive hammerhead ribozyme into the guide RNAs targeting the HEK-3 and FANCF sites. Indeed, these agRNAs resulted in small molecule-controlled base editing. The FANCF site exhibited 1.0–1.2% base editing with the theophylline-agRNA in the absence of theophylline (Fig. 4e). The addition of 4 mM theophylline increased the editing to 3.6–3.9% (Fig. 4e). In contrast, a much lower dose of guide RNA plasmid was required for the HEK-3 site to achieve base editing. With 2 ng guide RNA plasmid and 200 ng BE3 plasmid, 1.7–2.1% base editing was observed at the HEK-3 locus (Fig. 4f). The presence of 4 mM theophylline raised base editing efficiency to 4.2–5.8% (Fig. 4f). By increasing the dose of guide RNA and BE3 plasmid, as high as 36% base editing efficiency could be achieved in the presence of theophylline with two- to threefold ligand dependence (Fig. 4g,h). To test the ligand specificity of small molecule-controlled base editing, we measured the response of theophylline-agRNA-mediated base editing on the FANCF site in the presence of 3-methylxanthine, a close analogue of theophylline lacking one methyl group. At a ligand concentration of 4 mM, the theophylline-agRNA responded twofold less strongly to 3-methylxanthine than to theophylline (Supplementary Fig. 9). This lower response to 3-methylxanthine is consistent with the known preference of the theophylline-dependent ribozyme for theophylline over 3-methylxanthine^30^. Collectively, these results demonstrate the ability of agRNAs to confer small molecule-dependence on base editing at endogenous loci in human cells.

## Discussion

Aptazymes have been applied to control biological processes in various contexts, such as gene expression in prokaryotes and miRNA processing in mammalian cells^23^,^24^. By using aptazymes to remove blocking sequences that abrogate essential guide RNA structures, we have developed a set of small molecule-responsive agRNAs that enable exogenous control over genome engineering in mammalian cells using strategies that are complementary to other conditional Cas9 variants. In addition, small molecule-dependent guide RNAs have been reported in CRISPR ‘signal conductors’ to control gene expression for constructing logic gates and genetic circuits^31^. Our work serves as the first example to our knowledge of small molecule-controlled genome editing in mammalian cells achieved through guide RNA engineering. The non-cleavable theophylline-agRNA ((d)theophylline-agRNA) did not show any response to the small molecule for both genome editing and gene activation, strongly suggesting that the cleavage activity of the ribozyme is crucial for guide RNA activation. Because the most widely used sgRNA, that derived from *Streptococcus pyogenes*, has a length >100 nucleotides and contains multiple regions susceptible to secondary structural changes^19^, our strategy of triggering the self-cleavage of the blocking region from the guide RNA minimizes possible interference with guide RNA activity from the regulatory element since the activated agRNA closely resembles that of a canonical sgRNA.

Both nature and researchers have evolved hundreds of RNA aptamers that bind a wide variety of ligands^32^,^33^. By incorporating these aptamers into the agRNA architecture, agRNAs could potentially be constructed to respond to many different molecules of interest. As ligand specificities of aptamers are generally high, agRNAs have the potential to be multiplexed for complicated applications in which precise temporal and spatial control of multiple genome engineering events is required. The successful engineering of artificial guide RNAs to be ligand-responsive raises the possibility that naturally occurring CRISPR components may already use small-molecule regulation.

Although promising, this first generation of agRNAs exhibit detectable off-state activities in the absence of ligands, which may limit their applications in situations that require minimal off-state activity. We attribute the off-state activity of these agRNAs to two origins. First, aptazymes are typically constructed by fusing an aptamer to the ribozyme using a library of stem-loop variants, followed by screening for desired ligand dependence. Most of the aptazymes developed using this strategy are not completely inactivated in the absence of ligand. Second, the agRNA contains separate regulatory domain and functional domain, both with over 100 nucleotides. It is possible that higher degrees of ligand responsiveness would benefit from additional randomization and selection to optimize agRNA secondary structure and avoid inter-domain interference that increases background or decreases activity in the presence of ligand. Various parameters including communication between the aptamer and the ribozyme, interaction between the aptazyme and the guide RNA, hybridization of the blocking sequence and the spacer, as well as the binding affinity of guide RNA backbone to Cas9 in the presence or absence of the regulatory domain could be optimized in future generations of agRNAs.

## Methods

### Construction of guide RNA and reporter plasmids

Plasmids that overexpress Cas9 (pJDS246) and dCas9-VPR (pAWG-dCas9-VPR^4^) were used for mammalian cell experiments. The guide RNA plasmids were constructed based on the previously reported pFYF1320 plasmid^34^ and sequences of the guide RNAs were provided in Supplementary Table 1. The GFP and RFP reporter plasmids were constructed by swapping the promoter and the reporter gene from a previously reported plasmid (TF reporter for gRNA-AAVS1_T1, Addgene #47320 ref. 3) and the modified sequences were listed in Supplementary Table 2. Plasmids constructed in this study are available from Addgene.

### *In vitro* DNA cleavage assay

SpCas9 protein was purified as previously described^1^,^35^. Briefly, BL21 Star (DE3) competent *E. coli* cells were transformed with the plasmid encoding the bacterial codon-optimized Cas9. A single colony was picked and grown at 37 °C with vigorous shaking overnight. The dense bacterial culture was inoculated 1:200 into fresh LB media and grown at 37 °C until the OD600 reached 0.8. Isopropyl-β-D-1-thiogalactopyranoside (IPTG, GoldBio) was added to a final concentration of 0.5 mM and the culture was incubated for 16 h with shaking at 18 °C. The Cas9 protein was purified using Ni-NTA followed by cation exchange chromatography. Guide RNAs were transcribed using a T7 High Yield RNA Synthesis Kit (NEB) following the manufacturer’s protocol, and purified with the E.Z.N.A. PF miRNA Isolation Kit (Omega Bio-tek, Inc.). The target dsDNA (full GFP gene) was amplified by polymerase chain reaction and purified using QIAquick PCR Purification Kit (Qiagen). For the cleavage reaction, 10 nM of the target DNA was incubated with 100 nM of the guide RNA in the presence of 100 nM Cas9 protein in a Cas9 DNA cleavage buffer (150 mM KCl, 10 mM MgCl_2_, 0.5 mM DTT, 0.1 mM EDTA, 20 mM HEPES pH 7.5). *In vitro* editing reactions were incubated at 37 °C for 1 h for sgRNA and its variants and the reactions for crRNA and tracrRNA and their variants were incubated at 37 °C for 5 h before being stopped with 6 × DNA loading buffer and analysed by non-denaturing agarose gel electrophoresis.

### Editing of GFP gene in HEK293 cells

HEK293 cells with integrated GFP genes (GenTarget Inc.) were cultured in 48-well plates (∼40,000 cells seeded per well) in DMEM plus GlutaMAX (Life Technologies) with 10% FBS. Plasmids were transfected ∼20 h after plating when cells reached ∼70% confluence. To edit the GFP genes in HEK293 cells, 100 ng of Cas9 plasmid and 2 ng of guide RNA plasmid were transfected in each well using 1.2 μl Lipofectamine 2000 (Life Technologies) following the manufacturer’s protocol. To distinguish transfected cells from non-transfected cells, 2 ng of an iRFP expression plasmid was co-transfected. A stock solution of 100 mM theophylline was made in 60 mM NaOH and supplied to cells 6 h after plasmid transfection at designed concentrations. The cells were incubated for additional 7 days before analysis and media was changed at day 3, 5 and 6. Fresh theophylline was supplied immediately at the same concentration as in the initial condition each time the media was changed. GFP fluorescence was quantified by flow cytometry and the transfected live population was gated by iRFP signal.

### Transcriptional activation in HEK293T cells

Similar cell culture procedures were used for HEK293T cells as described above for HEK293-GFP cells. To each well in a 48-well plate, 200 ng of dCas9-VPR plasmid, 0.5 ng of guide RNA plasmid and 60 ng of reporter plasmid were transfected using 1.2 μl Lipofectamine 2,000. To distinguish transfected cells from non-transfected cells, 0.2 ng of an iRFP expression plasmid was co-transfected. A stock solution of 20 mM guanine was made in 60 mM NaOH and supplied to cells 6 h after plasmid transfection at designed concentrations. Cells were harvested 20 h after transfection. The GFP or RFP fluorescence was quantified by flow cytometry and the transfected live population was gated by iRFP signal.

### Genome editing and base editing in HEK293T cells

Similar cell culture procedures were used for HEK293T cells as described above for HEK293-GFP cells. A modified guide RNA plasmid containing an iRFP gene was used to differentiate transfected cells from untransfected ones when necessary. To each well in a 48-well plate, 400 ng of Cas9 or BE3 plasmid and 40 ng of guide RNA plasmid were transfected using 1.25 μl Lipofectamine 2000. Cells were collected 3 days after transfection. To test the small-molecule response, HEK293T cells were plated in a 24-well plate (100,000 cells seeded in each well) and BE3 and guide RNA plasmids were transfected 24 h after plating when cells reached ∼70% confluence. For the FANCF site, 800 ng BE3 and 80 ng guide RNA plasmids were transfected. For the HEK-3 site, 200, 400 or 800 ng of BE3 and 2, 10 or 80 ng of guide RNA plasmids were transfected, respectively. Each transfection used 2.5 μl Lipofectamine 2000. Stock solutions of 100 mM theophylline and 100 mM 3-methylxanthine were made in 100 mM NaOH and supplied to cells 6 h after plasmid transfection at specified concentrations. Cells were cultured for 3 days after transfection before sorting by flow cytometry. Indel formation and base editing were quantified by PCR amplification of the endogenous genomic loci of interest and analysis using high-throughput DNA sequencing. Sequencing alignment was performed using Burrows–Wheeler Aligner^36^.

### Data availability

High-throughput sequencing data are available from the NCBI Sequence Read Archive database under accession code SRP106902. All the other data are available from the authors upon reasonable request.

## Additional information

**How to cite this article:** Tang, W. *et al*. Aptazyme-embedded guide RNAs enable ligand-responsive genome editing and transcriptional activation. *Nat. Commun.* **8**, 15939 doi: 10.1038/ncomms15939 (2017).

**Publisher’s note**: Springer Nature remains neutral with regard to jurisdictional claims in published maps and institutional affiliations.

## Supplementary Material

Supplementary Information
