# Supplementary material for: Aptazyme-embedded guide RNAs enable ligand-responsive genome editing and transcriptional activation
Source: Nat Commun. 2017 Jun 28;8:15939. doi: 10.1038/ncomms15939 (PMC5493748; doi:10.1038/ncomms15939)
Supplement: Supplementary Information [file ncomms15939-s1.pdf]

File name: Supplementary Information
Description: Supplementary figures and supplementary tables.

Supplementary Table 1 | Sequences of guide RNAs. The spacer sequences are in bold and the blocking sequences are underlined. Aptamer units in agRNAs are in italics and the catalytic inactivating mutation in the hammerhead ribozyme is marked in red.

construct	sequence	plasmid
sgRNA-GFP	**GGGCACGGGCAGCTTGCCGG**GTTTTAGAGCTAGAAATA<br>GCAAGTTAAAATAAGGCTAGTCCGTTATCAACTTGAAAA<br>AGTGGCACCGAGTCGGTGCTTTTTTT	pFYF1320
5'-spacer-blocked sgRNA-GFP	GGTGCCCGTAGTAGTAGTAGCAACCACGTCGCGTCCGTA<br>GTAGTAGTA**GGGCACGGGCAGCTTGCCGG**GTTTTAGAG<br>CTAGAAATAGCAAGTTAAAATAAGGCTAGTCCGTTATCA<br>ACTTGAAAAAGTGGCACCGAGTCGGTGCTTTTTTT	-
3'-blocked crRNA-GFP	**GGGCACGGGCAGCTTGCCGG**GTTTTAGAGCTATGCTGT<br>TTTGGATGGCAACCACGTCGCGTCCTGTAGTAGTATGATA<br>GCTCTAA	-
5'-blocked tracrRNA	GGATTTTAACTTTAGTAGTAGTAGGCAACCACGTCGCGTC<br>CTAGTAGGCATAGCAAGTTAAAATAAGGCTAGTCCGTTAT<br>CAACTTGAAAAAGTGGCACCGAGTCGGTGCTTTTTTT	-
processed HHR-sgRNA-GFP	GCTGGATTCCAC**GGGCACGGGCAGCTTGCCGG**GTTTTA<br>GAGCTAGAAATAGCAAGTTAAAATAAGGCTAGTCCGTTA<br>TCAACTTGAAAAAGTGGCACCGAGTCGGTGCTTTTTTT	pWT016g
HHR-bsgRNA-GFP (10 nt blocker)	GCCCGTGCCCGGTACATCCAGCTGATGAGTCCCAAATAG<br>GACGAAACGCGCTTCGGTGCGTCCTGGATTCCAC**GGGCA**<br>**CGGGCAGCTTGCCGG**GTTTTAGAGCTAGAAATAGCAAG<br>TTAAAATAAGGCTAGTCCGTTATCAACTTGAAAAAGTGGC<br>ACCGAGTCGGTGCTTTTTTT	pWT016f
dHHR-bsgRNA-GFP (10 nt blocker)	GCCCGTGCCCGGTACATCCAGCTGATGAGTCCCAAATAG<br>GACGAGACGCGCTTCGGTGCGTCCTGGATTCCAC**GGGCA**<br>**CGGGCAGCTTGCCGG**GTTTTAGAGCTAGAAATAGCAAG<br>TTAAAATAAGGCTAGTCCGTTATCAACTTGAAAAAGTGGC<br>ACCGAGTCGGTGCTTTTTTT	pWT016e
HHR-bsgRNA-GFP (13 nt blocker)	GCTGCCCGTGCCCGGTACATCCAGCTGATGAGTCCCAAAT<br>AGGACGAAACGCGCTTCGGTGCGTCCTGGATTCCAC**GGG**<br>**CACGGGCAGCTTGCCGG**GTTTTAGAGCTAGAAATAGCA<br>AGTTAAAATAAGGCTAGTCCGTTATCAACTTGAAAAAGT<br>GGCACCGAGTCGGTGCTTTTTTT	pWT016a
dHHR-bsgRNA-GFP (13 nt blocker)	GCTGCCCGTGCCCGGTACATCCAGCTGATGAGTCCCAAAT<br>AGGACGAGACGCGCTTCGGTGCGTCCTGGATTCCAC**GGG**<br>**CACGGGCAGCTTGCCGG**GTTTTAGAGCTAGAAATAGCA<br>AGTTAAAATAAGGCTAGTCCGTTATCAACTTGAAAAAGT<br>GGCACCGAGTCGGTGCTTTTTTT	pWT016b
HHR-bsgRNA-GFP (17 nt blocker)	GCAAGCTGCCCGTGCCCGGTACATCCAGCTGATGAGTCCC<br>AAATAGGACGAAACGCGCTTCGGTGCGTCCTGGATTCCA<br>C**GGGCACGGGCAGCTTGCCGG**GTTTTAGAGCTAGAAAT<br>AGCAAGTTAAAATAAGGCTAGTCCGTTATCAACTTGAAA<br>AAGTGGCACCGAGTCGGTGCTTTTTTT	pWT016h

dHHR-bsgRNA-GFP (17 nt blocker)	GCAAGCTGCCCGTGCCCGGTACATCCAGCTGATGAGTCCCAAATAGGACGAGACGCGCTTCGGTGCGTCCTGGATTCCAC**GGGCACGGGCAGCTTGCCGG**GTTTTAGAGCTAGAAATAGCAAGTTAAAATAAGGCTAGTCCGTTATCAACTTGAAAAAGTGGCACCGAGTCGGTGCTTTTTTT	pWT016d
theophylline-agRNA-GFP	GCAAGCTGCCCGTGCCCGGTACATCCAGCTGATGAGTCCCAAATAGGACGAAA*TACATACCAGCCGAAAGGCCCTTGGCAGGTG*TCCTGGATTCCAC**GGGCACGGGCAGCTTGCCGG**GTTTTAGAGCTAGAAATAGCAAGTTAAAATAAGGCTAGTCCGTTATCAACTTGAAAAAGTGGCACCGAGTCGGTGCTTTTTTT	pWT016i
(d)theophylline-agRNA-GFP	GCAAGCTGCCCGTGCCCGGTACATCCAGCTGATGAGTCCCAAATAGGACGAGA*TACATACCAGCCGAAAGGCCCTTGGCAGGTG*TCCTGGATTCCAC**GGGCACGGGCAGCTTGCCGG**GTTTTAGAGCTAGAAATAGCAAGTTAAAATAAGGCTAGTCCGTTATCAACTTGAAAAAGTGGCACCGAGTCGGTGCTTTTTTT	pWT016l
guanine-agRNA (GFP activation)	GCAAGCTGCCCGTGCCCGGTACATCCAGCTGATGAGTCCCAAATAGGACGAAATAC*TATAATCGCGTGGATATGGCACGCAAGTTTCTACCGGGCACCGTAAATGTCCGACTA*GTGTCCTGGATTCCAC**GGGCACGGGCAGCTTGCCGG**GTTTTAGAGCTAGAAATAGCAAGTTAAAATAAGGCTAGTCCGTTATCAACTTGAAAAAGTGGCACCGAGTCGGTGCTTTTTTT	pWT029c
(d)guanine – agRNA (GFP activation)	GCAAGCTGCCCGTGCCCGGTACATCCAGCTGATGAGTCCCAAATAGGACGAGATAC*TATAATCGCGTGGATATGGCACGCAAGTTTCTACCGGGCACCGTAAATGTCCGACTA*GTGTCCTGGATTCCAC**GGGCACGGGCAGCTTGCCGG**GTTTTAGAGCTAGAAATAGCAAGTTAAAATAAGGCTAGTCCGTTATCAACTTGAAAAAGTGGCACCGAGTCGGTGCTTTTTTT	pWT029d
guanine-agRNA (GFP activation, 18 nt blocker with bulges)	GGCATGCTCCCCGTGACCGGTACATCCAGCTGATGAGTCCCAAATAGGACGAAATAC*TATAATCGCGTGGATATGGCACGCAAGTTTCTACCGGGCACCGTAAATGTCCGACTA*GTGTCCTGGATTCCAC**GGGCACGGGCAGCTTGCCGG**GTTTTAGAGCTAGAAATAGCAAGTTAAAATAAGGCTAGTCCGTTATCAACTTGAAAAAGTGGCACCGAGTCGGTGCTTTTTTT	pWT029g
(d)guanine-agRNA (GFP activation,18 nt blocker with bulges)	GGCATGCTCCCCGTGACCGGTACATCCAGCTGATGAGTCCCAAATAGGACGAGATAC*TATAATCGCGTGGATATGGCACGCAAGTTTCTACCGGGCACCGTAAATGTCCGACTA*GTGTCCTGGATTCCAC**GGGCACGGGCAGCTTGCCGG**GTTTTAGAGCTAGAAATAGCAAGTTAAAATAAGGCTAGTCCGTTATCAACTTGAAAAAGTGGCACCGAGTCGGTGCTTTTTTT	pWT029h
guanine-agRNA (19 nt blocker with	ACTGCGGGATGGAGGAGACGGTACATCCAGCTGATGAGTCCCAAATAGGACGAAATAC*TATAATCGCGTGGATATGGCACGCAAGTTTCTACCGGGCACCGTAAATGTCCGACTA*GTGTCCTGGATTCCAC**GTCCCCTCCACCCCACAGTG**GTTTTAGAGC	pWT049d

bulges, RFP activation)	TAGAAATAGCAAGTTAAAATAAGGCTAGTCCGTTATCAACTTGAAAAAGTGGCACCGAGTCGGTGCTTTTTTT	
(d)guanine-agRNA (19 nt blocker with bulges, RFP activation)	<u>ACTGCGGGATGGAGGAGAC</u>GGTACATCCAGCTGATGAGTCCCAAATAGGACGAGATAC*TATAATCGCGTGGATATGGCACGCAAGTTTCTACCGGGCACCGTAAATGTCCGACTA*GTGTCCTGGATTCCAC**GTCCCCTCCACCCCACAGTG**GTTTTAGAGCTAGAAATAGCAAGTTAAAATAAGGCTAGTCCGTTATCAACTTGAAAAAGTGGCACCGAGTCGGTGCTTTTTTT	pWT049e
sgRNA-HEK3	**GGCCCAGACTGAGCACGTGA**GTTTTAGAGCTAGAAATAGCAAGTTAAAATAAGGCTAGTCCGTTATCAACTTGAAAAAGTGGCACCGAGTCGGTGCTTTTTTT	pWT055g
sgRNA-FANCF	**GGAATCCCTTCTGCAGCACC**GTTTTAGAGCTAGAAATAGCAAGTTAAAATAAGGCTAGTCCGTTATCAACTTGAAAAAGTGGCACCGAGTCGGTGCTTTTTTT	pWT055f
sgRNA-EMX1	**GAGTCCGAGCAGAAGAAGAA**GTTTTAGAGCTAGAAATAGCAAGTTAAAATAAGGCTAGTCCGTTATCAACTTGAAAAAGTGGCACCGAGTCGGTGCTTTTTTT	pWT055e
sgRNA-HEK4	**GGCACTGCGGCTGGAGGTGG**GTTTTAGAGCTAGAAATAGCAAGTTAAAATAAGGCTAGTCCGTTATCAACTTGAAAAAGTGGCACCGAGTCGGTGCTTTTTTT	pWT055h
HHR-bsgRNA-HEK3	<u>CGTGCTCAGTCTGGGCC</u>GGTACATCCAGCTGATGAGTCCCAAATAGGACGAAACGCGCTTCGGTGCGTCCTGGATTCCAC**GGCCCAGACTGAGCACGTGA**GTTTTAGAGCTAGAAATAGCAAGTTAAAATAAGGCTAGTCCGTTATCAACTTGAAAAAGTGGCACCGAGTCGGTGCTTTTTTT	pWT057e
dHHR-bsgRNA-HEK3	<u>CGTGCTCAGTCTGGGCC</u>GGTACATCCAGCTGATGAGTCCCAAATAGGACGAGACGCGCTTCGGTGCGTCCTGGATTCCAC**GGCCCAGACTGAGCACGTGA**GTTTTAGAGCTAGAAATAGCAAGTTAAAATAAGGCTAGTCCGTTATCAACTTGAAAAAGTGGCACCGAGTCGGTGCTTTTTTT	pWT057f
HHR-bsgRNA-FANCF	<u>GCTGCAGAAGGGATTCC</u>GGTACATCCAGCTGATGAGTCCCAAATAGGACGAAACGCGCTTCGGTGCGTCCTGGATTCCAC**GGAATCCCTTCTGCAGCACC**GTTTTAGAGCTAGAAATAGCAAGTTAAAATAAGGCTAGTCCGTTATCAACTTGAAAAAGTGGCACCGAGTCGGTGCTTTTTTT	pWT057c
dHHR-bsgRNA-FANCF	<u>GCTGCAGAAGGGATTCC</u>GGTACATCCAGCTGATGAGTCCCAAATAGGACGAGACGCGCTTCGGTGCGTCCTGGATTCCAC**GGAATCCCTTCTGCAGCACC**GTTTTAGAGCTAGAAATAGCAAGTTAAAATAAGGCTAGTCCGTTATCAACTTGAAAAAGTGGCACCGAGTCGGTGCTTTTTTT	pWT057d
HHR-bsgRNA-EMX1	<u>GTTCTTCTGCTCGGACTC</u>GGTACATCCAGCTGATGAGTCCCAAATAGGACGAAACGCGCTTCGGTGCGTCCTGGATTCCAC**GAGTCCGAGCAGAAGAAGAA**GTTTTAGAGCTAGAAATAGCAAGTTAAAATAAGGCTAGTCCGTTATCAACTTGAAAAAGTGGCACCGAGTCGGTGCTTTTTTT	pWT057a

dHHR-bsgRNA-EMX1	GTTCTTCTGCTCGGACTCGGTACATCCAGCTGATGAGTCCCAAATAGGACGAGACGCGCTTCGGTGCGTCCTGGATTCCAC**GAGTCCGAGCAGAAGAAGAA**GTTTAGAGCTAGAAATAGCAAGTTAAAATAAGGCTAGTCCGTTATCAACTTGAAAAAGTGGCACCGAGTCGGTGCTTTTTTT	pWT057b
HHR-bsgRNA-HEK4	GCCTCCAGCCGCAGTGCCGGTACATCCAGCTGATGAGTCCCAAATAGGACGAAACGCGCTTCGGTGCGTCCTGGATTCCAC**GGCACTGCGGCTGGAGGTGG**GTTTAGAGCTAGAAATAGCAAGTTAAAATAAGGCTAGTCCGTTATCAACTTGAAAAAGTGGCACCGAGTCGGTGCTTTTTTT	pWT057g
dHHR-bsgRNA-HEK4	GCCTCCAGCCGCAGTGCCGGTACATCCAGCTGATGAGTCCCAAATAGGACGAGACGCGCTTCGGTGCGTCCTGGATTCCAC**GGCACTGCGGCTGGAGGTGG**GTTTAGAGCTAGAAATAGCAAGTTAAAATAAGGCTAGTCCGTTATCAACTTGAAAAAGTGGCACCGAGTCGGTGCTTTTTTT	pWT057h
theophylline-agRNA-HEK3	CGTGCTCAGTCTGGGCCGGTACATCCAGCTGATGAGTCCCAAATAGGACGAAA*TACATACCAGCCGAAAGGCCCTTGGCAGGTG*TCCTGGATTCCAC**GGCCCAGACTGAGCACGTGA**GTTTTAGAGCTAGAAATAGCAAGTTAAAATAAGGCTAGTCCGTTATCAACTTGAAAAAGTGGCACCGAGTCGGTGCTTTTTTT	pWT055c
theophylline-agRNA-FANCF	GCTGCAGAAGGGATTCCGGTACATCCAGCTGATGAGTCCCAAATAGGACGAAA*TACATACCAGCCGAAAGGCCCTTGGCAGGTG*TCCTGGATTCCAC**GGAATCCCTTCTGCAGCACC**GTTTTAGAGCTAGAAATAGCAAGTTAAAATAAGGCTAGTCCGTTATCAACTTGAAAAAGTGGCACCGAGTCGGTGCTTTTTTT	pWT055b

Supplementary Table 2 | Sequences of the engineered promoter and reporter genes. The protospacer sequences are in bold and the engineered promoter is underlined. The GFP and RFP genes are in italic.

construct	sequence	plasmid
GFP reporter	**GGGCACGGGCAGCTTGCCGG**GGGCGAGGTAGGCGTGTACGGTGGGAGGCCTATATAAGCAGAGCTCGTTTAGTGAACCGTCAGATCGCCTGGAGAATTCGCCACC*ATGGACTACAAGGATGACGACGATAAAACTTCCGGTGGCGGACTGGGTTCCACCCGTAAAGGTGAAGAACTGTTCACCGGTGTTGTTCCGATCCTGGTTGAACTGGACGGTGACGTTAACGGTCACAAATTCTCTGTTCGTGGTGAAGGTGAAGGTGACGCTACCAACGGTAAACTGACCCTGAAATTCATCTGCACCACCGGTAAACTGCCGGTTCCGTGGCCGACCCTGGTTACCACCCTGACCTACGGTGTTCAGTGCTTCGCTCGTTACCCGGACCACATGAAACAGCACGACTTCTTCAAATCTGCTATGCCGGAAGGTTACGTTCAGGAACGTACCATCTCTTTCAAAGACGACGGTACCTACAAAACCCGTGCTGAAGTTAAATTCGAAGGTGACACCCTGGTTAACCGTATCGAACTGAAAGGTATCGACTTCAAAGAAGACGGTAACATCCTGGGTCACAAACTGGAATACAACTTCAACTCTCACAACGTTTACATCACCGCTGACAAACAGAAAAACGGTATCAAAGCTAACTTCAAAATCCGTCACAACGTTGAAGACGGTTCTGTTCAGCTGGCTGACCACTACCAGCAGAA*	pWT015a

	CACCCCGATCGGTGACGGTCCGGTTCTGCTGCCGGACAACC<br>*ACTACCTGTCTACCCAGTCTGTTCTGTCTAAAGACCCGAACGA*<br>*AAAACGTGACCACATGGTTCTGCTGGAATTCGTTACCGCTGCT*<br>*GGTATCACCCACGGTATGGACGAACTGTACAAA*	
RFP reporter	**GTCCCCTCCACCCCACAGTG**GGGCGAGGTAGGCGTGTA<br>CGGTGGGAGGCCTATATAAGCAGAGCTCGTTTAGTGAAC<br>CGTCAGATCGCCTGGAGAATTCGCCACC*ATGGACTACAAG*<br>*GATGACGACGATAAAACTTCCGGTGGCGGACTGGGTTCCACC*<br>*GCGAGCAAGGGCGAGGAGGATAACATGGCCATCATCAAGGA*<br>*GTTCATGCGCTTCAAGGTGCACATGGAGGGCTCCGTGAACG*<br>*GCCACGAGTTCGAGATCGAGGGCGAGGGCGAGGGCCGCCC*<br>*CTACGAGGGCACCCAGACCGCCAAGCTGAAGGTGACCAAGG*<br>*GCGGCCCCCTGCCCTTCGCCTGGGACATCCTGTCCCCTCAG*<br>*TTCATGTACGGCTCCAAGGCCTACGTGAAGCACCCCGCCGAC*<br>*ATCCCCGACTACTTGAAGCTGTCCTTCCCCGAGGGCTTCAAG*<br>*TGGGAGCGCGTGATGAACTTCGAGGACGGCGGCGTGGTGAC*<br>*CGTGACCCAGGACTCCTCCCTACAGGACGGCGAGTTCATCTA*<br>*CAAGGTGAAGCTGCGCGGCACCAACTTCCCCTCCGACGGCC*<br>*CCGTAATGCAGAAGAAGACGATGGGCTGGGAGGCCTCCTCC*<br>*GAGCGGATGTACCCCGAGGACGGCGCCCTGAAGGGCGAGAT*<br>*CAAGCAGAGGCTGAAGCTGAAGGACGGCGGCCACTACGACG*<br>*CCGAGGTCAAGACCACCTACAAGGCCAAGAAGCCCGTGCAG*<br>*CTGCCCGGCGCCTACAACGTCAACATCAAGTTGGACATCACC*<br>*TCCCACAACGAGGACTACACCATCGTGGAACAGTACGAGCGC*<br>*GCCGAGGGCCGCCACTCCACCGGCGGCATGGACGAGCTGTA*<br>*CAAGGCCCGCGGTTAA*	pWT032a

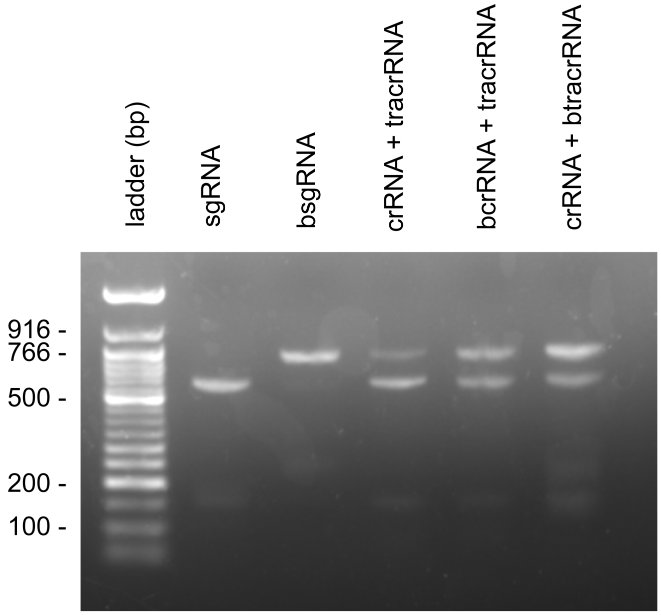

Supplementary Figure 1 | *In vitro* activity of unmodified guide RNAs and three blocked guide RNA variants (full gel).

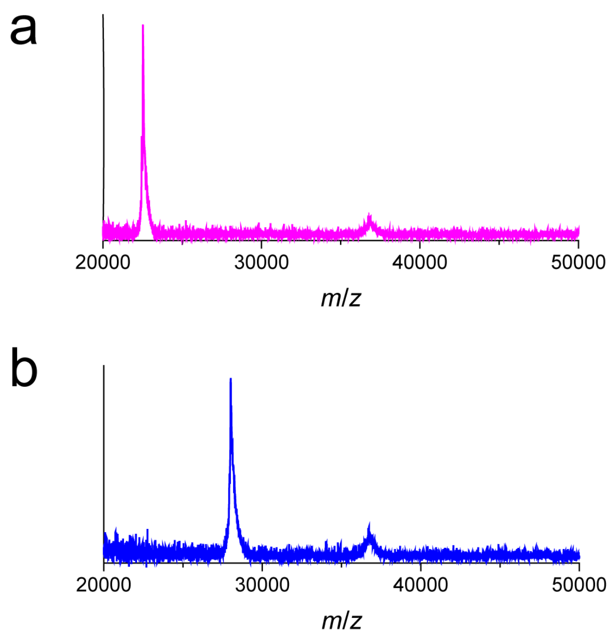

Supplementary Figure 2 | Matrix-assisted laser desorption ionization time-of-fight mass spectrometry analyses of cleaved HHR-bsgRNA (a) and theophylline-agRNA (b). Both the HHR-bsgRNA and theophylline-agRNA contain a 15 nt blocking sequence at the 5' end. HHR-bsgRNA, calculated M: 22,429 (5' fragment) and 36,783 (3' fragment), average mass; observed M + H^+: 22,439 and 36,808, average mass. Theophylline-agRNA, calculated M: 27,932 (5' fragment) and 36,783 (3' fragment), average mass; observed M + H^+: 28,006 and 36,734, average mass.

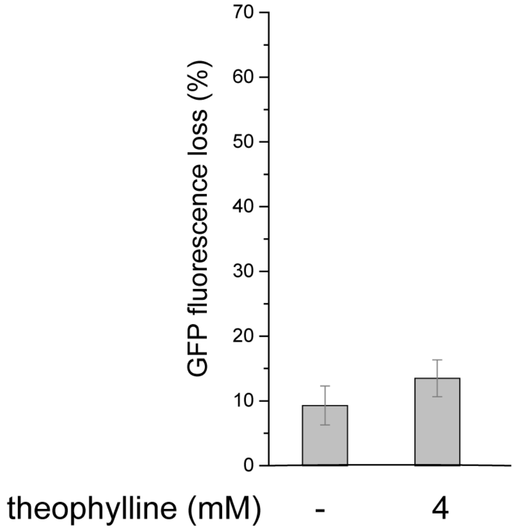

Supplementary Figure 3 | Genome-editing activity of (d)theophylline-agRNA in the presence and absence of 4 mM theophylline at endogenous GFP sites in HEK293-GFP cells.

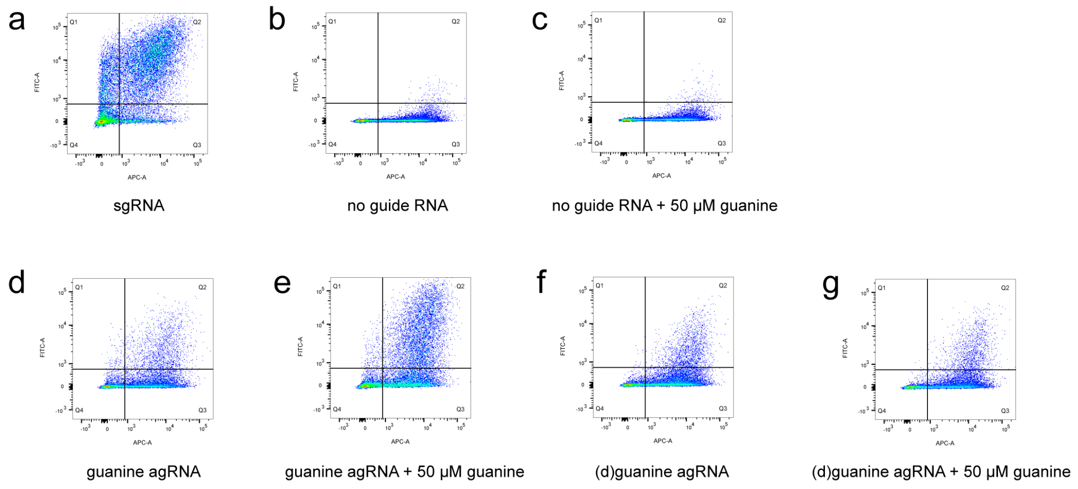

Supplementary Figure 4 | Representative flow cytometry scatter plots showing GFP activation in cells that were transfected with specified guide RNAs in the absence or presence of 50 μM guanine. APC signal was used to differentiate transfected cells using the iRFP fluorescence. Statistic results of the same set of experiments are shown in Fig. 3.

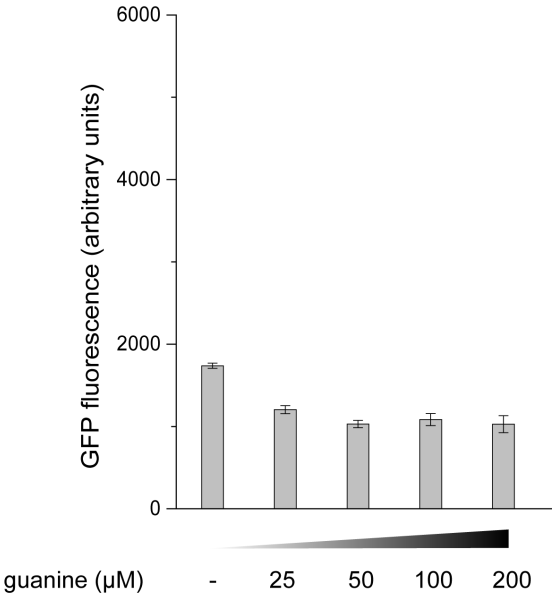

Supplementary Figure 5 | GFP activation in HEK293T cells by (d)guanine-agRNA in the presence of different concentrations of guanine.

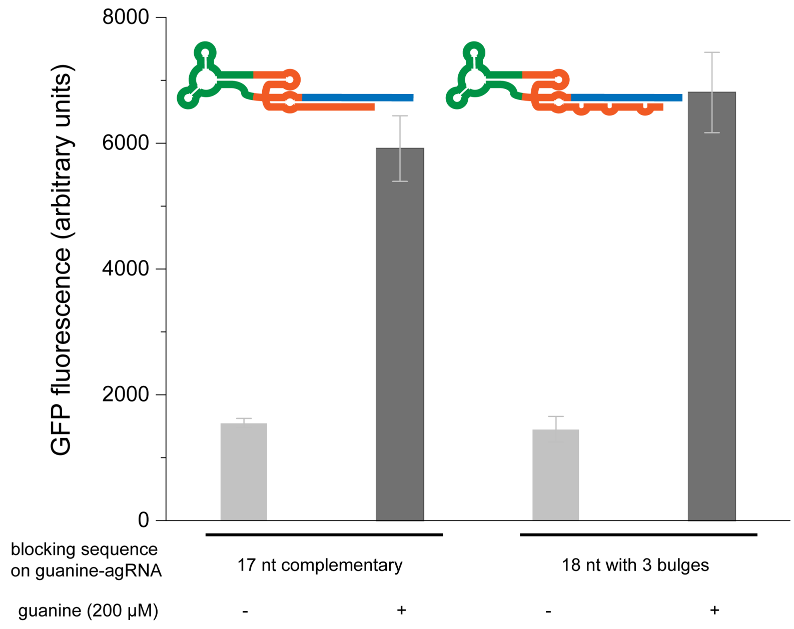

Supplementary Figure 6 | GFP activation activities of two architectures of guanine-agRNA in HEK293T cells. Different blocking sequences were used in the two guanine-agRNAs.

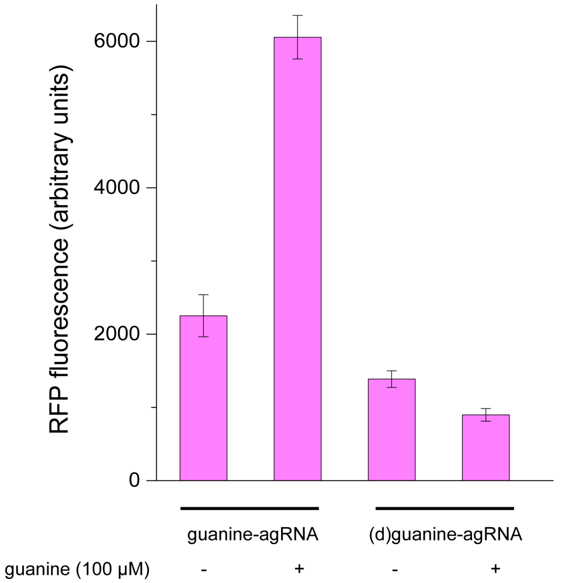

Supplementary Figure 7 | RFP activation by guanine-agRNA using a different spacer sequence in HEK293T cells. (d)Guanine-agRNA was included as a negative control.

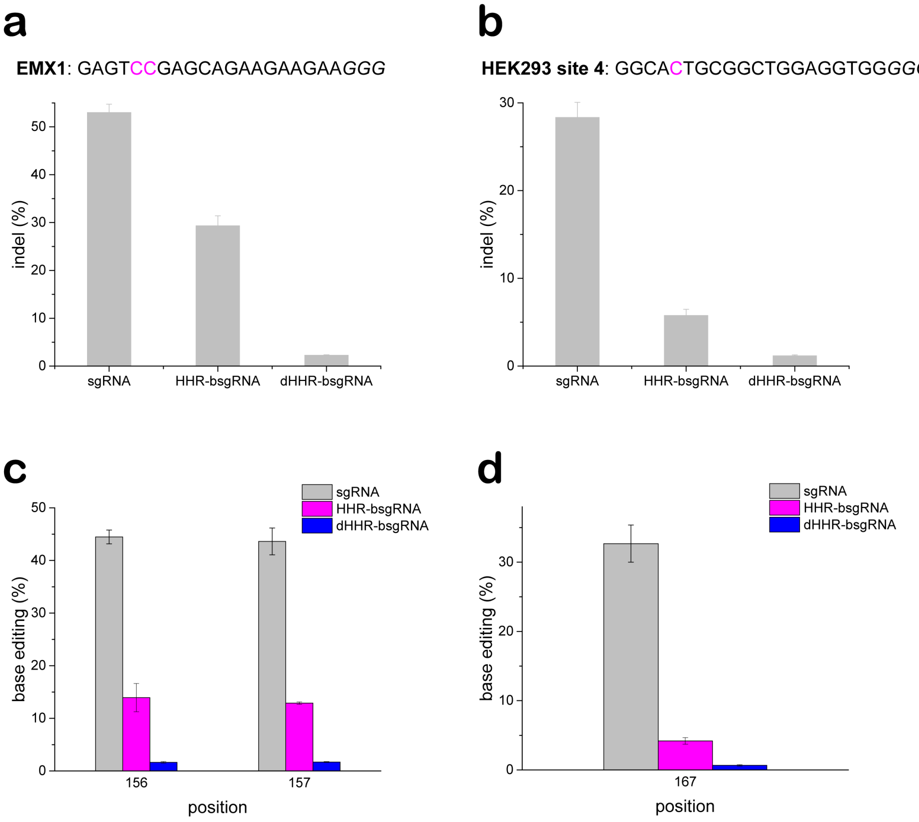

Supplementary Figure 8 | Nuclease-mediated indel formation and base editing by HHR-bsgRNA and dHHR-bsgRNA at the EMX-1 and HEK-4 sites.

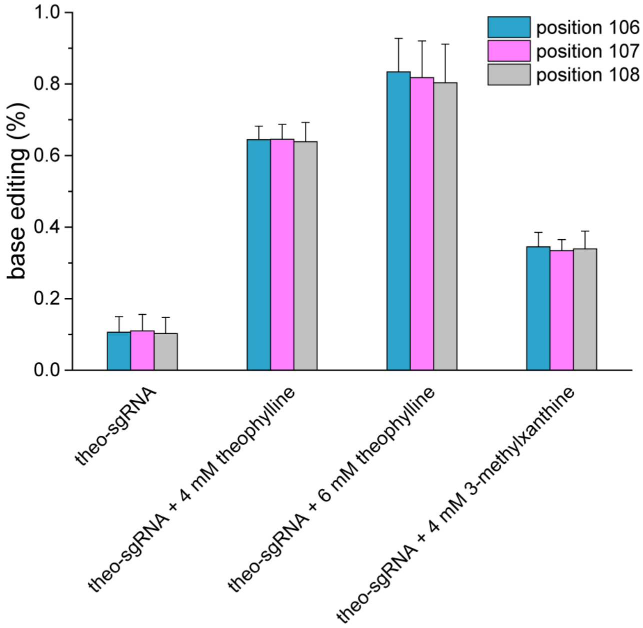

Supplementary Figure 9 | Base editing of theophylline-agRNA in the presence and absence of theophylline and 3-methylxanthine on the FANCF site in HEK293T cells.
